# Supplementary material for: Direct Optical Probing of Transverse Electric Mode in Graphene
Source: Sci Rep. 2016 Feb 22;6:21523. doi: 10.1038/srep21523 (PMC4761906; doi:10.1038/srep21523)
Supplement: Supplementary Information [file srep21523-s1.pdf]

Supplementary Information

Direct Optical Probing of Transverse Mode in Graphene

Sergey G. Menabde[1], Daniel R. Mason[1], Evgeny Kornev[2], Changhee Lee[2], and Namkyoo Park[1,]*

[1]*Photonic Systems Laboratory, School of ECE, Seoul National University, Seoul 151-744, Korea*

[2]*Organic Semiconductor Laboratory, School of ECE, Seoul National University, Seoul 151-744, Korea*

*nkpark@snu.ac.kr

1. Surface Graphene Conductivity at Finite Temperature

A complex sheet conductivity of a single graphene layer can be numerically calculated using the RPA in the local limit as follows[1]:

$$\sigma(\omega)=\ln\left[2\cosh\left(\frac{E_F}{2k_BT}\right)\right]\frac{2e^2k_BT}{\pi\hbar^2}\frac{i}{\omega+i/\tau}+\frac{e^2}{4\hbar}\left\{H\left(\omega/2\right)+\frac{4i\omega}{\pi}\int_0^\infty dx\frac{H\left(x\right)-H\left(\omega/2\right)}{\omega^2-4x^2}\right\}, \qquad \text{(S1)}$$

where $$H(x)=\frac{\sinh\left(\hbar x/k_BT\right)}{\cosh\left(E_F/k_BT\right)+\cosh\left(\hbar x/k_BT\right)},$$

where T is the temperature, k_B is the Boltzmann's constant, and $\tau = \mu E_F/ev_F^2$ is the electron-scattering relaxation time[2], with $\mu = 1\times10^4 \mathrm{cm^2(Vs)^{-1}}$ as the mobility, and $\upsilon_F = 1\times10^6\ \mathrm{ms^{-1}}$ as the Fermi velocity of charge carriers in graphene.

2. Multilayer Graphene Doping

We employ top-gated configuration for electrostatic graphene doping via the ionic liquid[3,4], using two graphene stripes as electrodes. When bias voltage is applied, a Debye layer in the ionic liquid of thickness d_{TG} ~1 nm is formed around the electrodes[4], acting as a parallel plate capacitor. Graphene electrode thus being one of the capacitor plates, so its charge carriers' concentration n can be related to the applied bias voltage V_g as follows[3]:

$$V_{TG}=\frac{\hbar\left|\upsilon_F\right|\sqrt{\pi n}}{e}+\frac{d_{TG}ne}{\varepsilon\varepsilon_0} \qquad \text{(S2)}$$

where ε is the dielectric constant of the ionic liquid, n is the carriers' concentration in graphene electrode. The Debye layer thickness in an electrolyte can be obtained as[5]:

$$d_{TG}=\sqrt{\frac{\varepsilon\varepsilon_0RT}{2F^2c_0}} \qquad \text{(S3)}$$

where R is the molar constant, $F = eN_A$ is the Faraday constant, and c_0 = 3.9 mol/l is the molar concentration of the 1-ethyl-3-methylimidazolium bis(trifluoromethylsulfonyl)imide ionic liquid[6,7]. Obtained value of the Debye layer $d_{TG} \approx 0.8$ nm is relatively small due to high electrolyte concentration of the ionic liquid, and comparable with thickness of several graphene layers, thus making difficult to predict actual charge distribution in real system of several layers where screening effects will occur. Therefore, as a first-order approximation, we assume that charge carriers' concentrations (Fermi energy) in all layers are equal, and the total effective carriers' concentration of the whole stack can be represented as $n = X \times n_{gr}$, where n_{gr} is concentration of a single layer, and X is some real number.

Considering the stack of graphene sheets as a single layer with Fermi energy as a function of effective carriers' concentration $E_F = \hbar|\upsilon_F|\sqrt{\pi n}$, following Eqs. S1-S3, we obtain values of Fermi level and RPA sheet conductivity of a single graphene layer as a function of applied bias voltage, which is demonstrated in Fig. S1. Interestingly, the assumption of $X = 3$ gives the exact match between experimentally observed bias voltages and conductivity values used for experimental results reconstruction by an electrodynamic model of the structure. In other words, assumed conductivity of each graphene layer in a five layer graphene stack can be taken as $n_{gr} = n/3$, where n is an effective concentration in multilayer graphene treated as a single layer. Then, setting equal the effective concentration in the electrodynamic model (EM) and the top gated (TG) doping model, for a single layer in the stack of five we can speculate that $n_{gr}^{EM} = 3/5 \times n_{gr}^{TG}$. This may indicate that actual doping levels in conducted experiments are ~40% lower than that theoretically obtained from Eqs. S2 and S3.

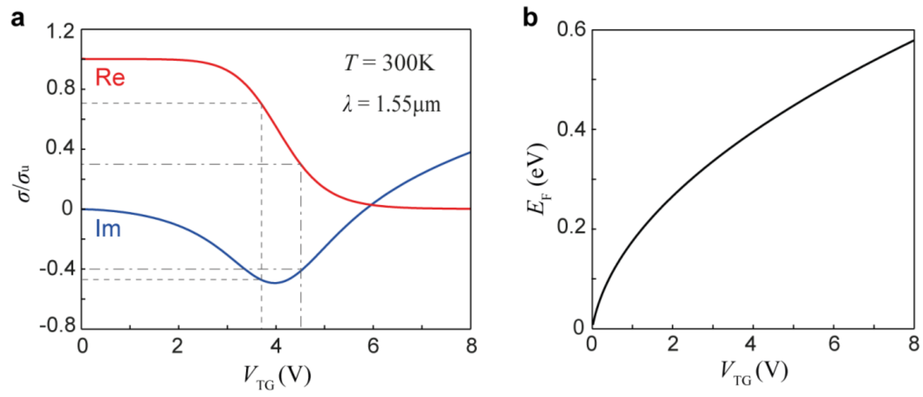

Figure S1. **a** RPA sheet conductivity of single layer graphene as a function of bias voltage; carriers' concentration of each single layer is assumed uniform through the five layer stack, and its value $n_{gr} = n/3$. Dot-dashed lines show real and imaginary parts of conductivity according to the experiment 1 reconstruction (model case 1 in main text; V_{TG} = 3.8 V, $\sigma_2 = 0.3\sigma_u - i0.4\sigma_u$), and dashed lines show that for the experiment 2 reconstruction (model case 2 in main text; V_{TG} = 4.5 V, $\sigma_2 = 0.7\sigma_u - i0.47\sigma_u$). **b** Fermi energy of a single graphene layer as a function of bias voltage for the same assumption as in **a**.

Our successful example of multilayer graphene doping requires more close study of the subject since multilayer graphene systems generally are able to provide significantly stronger electrodynamic response comparing with that built on a single graphene layer.

References

1. Falkovsky, L. A. Optical properties of graphene. *Journal of Physics: Conference Series* **129**, 012004 (2008).
2. Jablan, M., Buljan, H. & Soljačić, M. Plasmonics in graphene at infrared frequencies. *Phys.Rev. B* **80**, 245435 (2009).
3. Das, A. *et al*. Monitoring dopants by Raman scattering in an electrochemically top-gated graphene transistor. *Nature Nanotech.* **3**, 210-215 (2008).

4. Lu, C., Fu, Q., Huang, S. & Liu, J. Polymer electrolyte-gated carbon nanotube field-effect transistor. *Nano Lett.* **4**, 623-627 (2004).
5. Russel, W.B., Saville, D.A. & Schowalter, W. R. *Colloidal Dispersions* (Cambridge University Press, 1989).
6. McEwen, A. B., Ngo, H. L., LeCompte, K. & Goldman, J. L. Electrochemical properties of imidazolium salt electrolytes for electrochemical capacitor applications. *J. Electrochem. Soc.* **146**, 1687-1695 (1999).
7. Ohno, H. *Colloidal Dispersions Electrochemical Aspects of Ionic Liquids* Ch. 5 (John Wiley & Sons, New Jersey, 2011).
